# High Dental Caries among Adults Aged 35 to 44 Years: Case-Control Study of Distal and Proximal Factors

**DOI:** 10.3390/ijerph10062401

**Published:** 2013-06-07

**Authors:** Simone M. Costa, Mara Vasconcelos, Mauro H. N. G. Abreu

**Affiliations:** 1 Department of Dentistry, Universidade Estadual of Montes Claros, Unimontes Montes Claros, Minas Gerais, Av Dr Ruy Braga, Campus Universitário Professor Darcy Ribeiro, Prédio 6, Centro de Ciências Biológicas e da Saúde, Vila Mauricéia, Montes Claros, Minas Gerais 39.401-089, Brazil; E-Mail: smelocosta@gmail.com; 2 Department of Community and Preventive Dentistry, Universidade Federal of Minas Gerais, Belo Horizonte, Av. Antônio Carlos, 6627 Belo Horizonte, Minas Gerais 31270901, Brazil; E-Mail: maravas@uol.com.br

**Keywords:** oral health, dental caries, adults, epidemiology, socioeconomic status

## Abstract

The aim of this study was to determine whether a high degree of dental caries severity is associated with the distal and proximal determinants of caries in a group of Brazilian adults aged 35 to 44 years. A population-based case-control study was conducted using two groups—a case group with high caries severity (DMFT ≥ 14) and a control group without high caries severity (DMFT < 14). The sample comprised adults from metropolitan Belo Horizonte, Brazil (180 cases and 180 controls matched for gender and age). The exam was performed by calibrated dentists using the DMFT index. The statistical analysis used the Mann-Whitney test and bivariate and multivariate logistic regression (the conditional backward stepwise method). The mean DMFT was 8.4 ± 3.9 in the control group and 20.1 ± 4.5 in the case group. High caries severity was associated with regular visits to the dentist, low income, use of private/supplementary dental service and not petitioning the authorities for community benefits. The results of the study underscore the importance of considering distal and proximal factors in the assessment of the severity of dental caries. Greater caries severity persists among low-income families and among groups with a low degree of social cohesion.

## 1. Introduction

Case-studies are the most frequently undertaken type of analytical epidemiological study. For rare outcomes, they are the only practical study method. Case-control studies should be useful for common diseases [1,2]. The prevalence of dental caries among adults is high throughout the World [3]. A greater severity of dental caries may be associated with social, economic and individual determinants [4]. The conceptual model proposed by Petersen [4] to explain the severity of caries classifies the determinant factors as either distal or proximal. The distal level is related to socio-environmental factors and to the availability of oral health services, whereas the proximal level is related to modifiable behavior, such as oral hygiene practices, dietary habits, life style and the use of oral health services. 

Case-control studies of dental caries in adults aged 35 to 44 years are limited. A recent systematic review on the social determinants of dental caries in adults did not identify a case-control study [5]. A recent cross-sectional study of an identical population identified the importance of the social determinants involved in the health-illness process for epidemiological studies of dental caries [6]. Case-control studies can be useful for generating hypotheses that can be studied via a prospective cohort [1], which is remarkably rare in the epidemiology of dental caries in adults [5]. Researchers have not been concerned with case-control studies on dental caries in adults and with the potential determinants of the severity of this disease in case control studies, such as socioeconomic and behavioral factors. Understanding the determinants of dental caries in adults, with a range of distal and proximal variables, could provide evidence for the formulation of new policies designed to reduce social and economic inequalities. 

The aim of this study was to determine whether a high degree of dental caries severity is associated with distal and proximal determinants in a group of urban Brazilian adults aged 35 to 44 years. The hypothesis of this study is that a high degree of dental caries severity in adults is associated with the distal and proximal determinants of this outcome. 

## 2. Methods

This study is part of a broader-scoped cross-sectional study titled “Oral Health among Adults in Metropolitan Belo Horizonte (urban areas): Objective and Subjective Aspects”. The study was conducted in 2010 with adults aged 35 to 44 years, who were randomly selected from municipalities, districts/blocks and homes. The participants were selected using two- or three-stage cluster sampling: a random sampling of districts and blocks (municipalities ≥ 50,000 inhabitants) or of blocks (municipalities < 50,000 inhabitants) and homes. The sample was composed of residents from seven municipalities in metropolitan Belo Horizonte (southeastern Brazil), which is an industrialized city with an urban population of 2,375,151 [7]. The participation rate was 98.5%. The details of the study methods are available in a previous publication [6].

A population-based case-control study, which is nested within this cross-sectional survey, was conducted using two groups—a case group with high caries severity (DMFT ≥ 14) and a control group without high caries severity (DMFT < 14). The criterion for the selection of the cases was based on the following categories for the global occurrence of caries. Very low: <5; low: 5–8.9; moderate: 9–13.9; and high: >13.9 [3,8]. Brazil was ranked with high caries severity (DMFT > 13.9) in adults (35 to 44 years), and in this survey, the control group was represented by the categories very low, low and moderate. 

The sample size was calculated using a combined set of cases and controls, with one control individually matched for each case. The data from a pilot study (20 cases and 20 controls not included in the main sample) indicated that the probability of exposure (low monthly household income) among the controls was 0.1 and that the correlation coefficient for exposure among the cases and controls combined was 0.16; the odds ratio (OR) for caries among the exposed individuals in relation to the non-exposed individuals was 2.533. The sample size was calculated as 180 cases and 180 controls to reject the null hypothesis of an OR equal to 1 with an 80% power. The Power and Sample Size Calculation software program (version 3.0, Dupont W.D., Plummer W.D., Nashville, TN, USA) was used for this calculation. The groups were individually matched for age and gender and maintained the proportionality of the various municipalities evaluated. The probability of a type I error associated with the null hypothesis test was 0.05. 

Five examiners underwent a calibration process. The intra-examiner and inter-examiner Kappa values were 0.80 to 1.00 and 0.81 to 0.92, respectively. Clinical exams were performed under natural light in the home of each participant with the aid of a mouth mirror and a periodontal probe, following the recommendations of the WHO. The DMFT (number of decayed, missing and filled teeth) index was used [9]. The dependent variable was the severity of dental caries. The independent variables are listed in Table 1. The selection of the independent variables was based on the conceptual model of distal and proximal determinants for the outcome (severity of dental caries) proposed by Petersen [3,4].

Based on the literature, a questionnaire was designed for the collection of the independent variables [9,10,11,12]. Although not formally validated, this questionnaire was tested in a pilot study to determine the comprehension level of adults. The test-retest method was used to assess the responses of 25 adults on two occasions. The concordance between the responses upon the two administrations of the questionnaire was determined, and a high degree of reproducibility was demonstrated. A questionnaire is more reliable when it produces identical responses on different occasions [13]. The independent variables were dichotomized (Table 1).

Statistical analysis was performed using the Statistical Package for Social Sciences (SPSS 18.0), with the level of significance set to 5% (*p* < 0.05). The Mann-Whitney test was used to compare the DMFT indices between the control and case groups. Bivariate and multivariate conditional logistic regression analysis (the backward stepwise method) was used, considering 95% confidence intervals (95% CI) [14]. All of the variables with a *p*-value ≤ 0.20 in the bivariate analysis were incorporated into the multivariate analysis; those variables subsequently achieving a *p*-value < 0.05 were considered significant and remained in the final model [15].

This study received approval from the Human Research Ethics Committee of the Universidade Federal de Minas Gerais (Brazil) under process number 096/09. All participants consented to a clinical examination and an oral interview.

## 3. Results

**Figure 1:**
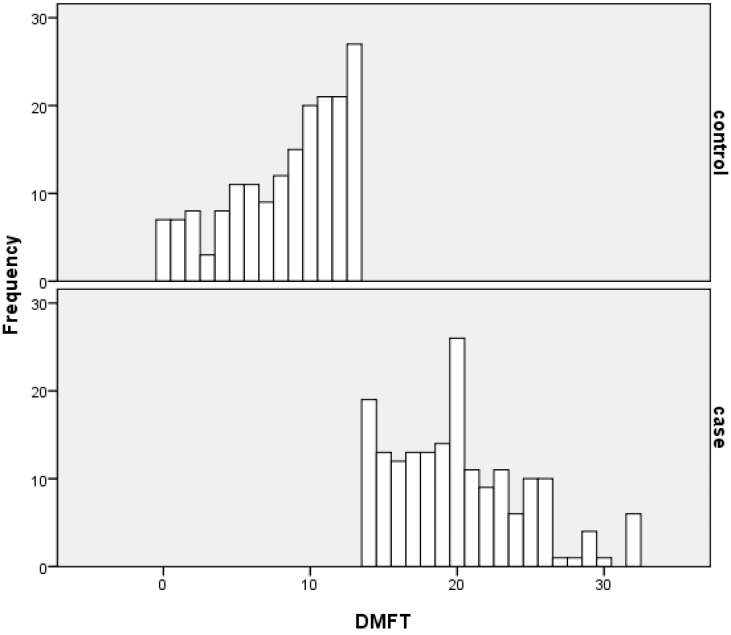
Distribution of DMFT in the case and control groups.

A total of 360 adults participated in this study (180 cases and 180 controls matched for age and gender). Figure 1 displays the percentage distribution of the DMFT in the groups. The mean DMFT index was 8.4 ± 3.9 in the control group and 20.1 ± 4.5 in the case group (*p* < 0.001). The decayed component contributed to 10.7% and 5.1% of the total DMFT in the control group and in the case group, respectively. The contribution of the missing component to the total DMFT was 30.7% and 34.4% in the control group and in the case group, respectively. The filled component was 58.6% and 60.5% in the control group and in the case group, respectively.

Regarding the distal determinants of dental caries severity, the entire sample lived in locations with proper basic sanitation (connection to a public water supply, connection to a sewage system and trash collection). The majority (97.2%) had at least one year of schooling, did not participate in groups (78.6%), was willing to dedicate time to community actions (90.4%), did not participate in community meetings (81.1%), was dissatisfied with access to healthcare services (60.6%), used private/supplementary dental services more than public services (71.8%), had no problems making an appointment (73.2%), knew individuals willing to lend them money (62.8%), did not take illicit drugs (98.6%) and felt that they had the power to make decisions that could change the direction of their lives (75.3%) (Table 1).

**Table 1:** Distal factors associated with high dental caries in adults, Brazil, 2010.

Distal factors		Cases (DMFT ≥ 14) n = 180 (%)	Controls (DMFT < 14) n = 180 (%)	Unadjusted odds ratio (95% CI)	Adjusted odds ratio (95% CI)
**Health system and oral health services**					
Satisfaction with access to services *					
	Yes	69 (38.5)	72 (40.2)	1	
	No	110 (61.5)	107 (59.8)	0.9 (0.6–1.4)	
Type of dental service used most *					
	Private/supplementary	138 (77.1)	116 (66.3)	1.7 (1.1–2.7)	2.3 (1.2–4.3)
	Public	41 (22.9)	59 (33.7)	1	1
Problem scheduling an appointment *					
	Yes	31 (22.8)	43 (30.7)	1	
	No	105 (77.2)	97 (69.3)	1.5 (0.9–2.6)	
**Socio-cultural determinants**					
Monthly household income		86 (47.8)	110 (61.1)	1	1
	>R$1,020 or US$600	94 (52.2)	70 (38.9)	1.7 (1.1–2.6)	2.2 (1.3–3.9)
	≤R$1,020 or US$600				
Schooling *					
	Literate	176 (98.9)	172 (95.6)	1	
	Illiterate	2 (1.1)	8 (4.4)	0.2 (0.1–1.2)	
**Social support network**					
Participation in groups					
	Yes	41 (22.8)	36 (20.0)	1	
	No	139 (77.2)	144 (80.0)	0.9 (0.5–1.4)	
Willing to dedicate time to community activities *					
	Yes	156 (88.1)	166 (92.7)	1	
	No	21 (11.9)	13 (7.3)	1.7 (0.8–3.6)	
Participation in neighborhood meetings *					
	Yes	32 (17.8)	36 (20.1)	1	
	No	148 (82.2)	143 (79.9)	1.2 (0.7–2.0)	
Have persons willing to assist financially					
	Yes	112 (62.2)	114 (63.3)	1	
	No	68 (37.8)	66 (36.7)	1.1 (0.7–1.6)	
Feel safe at home *					
	Yes	64 (35.8)	62 (34.4)	1	
	No	115 (64.2)	118 (65.6)	0.9 (0.6–1.5)	
Neighborhood petitioned the authorities in previous year *					
	Yes	66 (39.5)	94 (53.7)	1	1
	No	101 (60.5)	81 (46.3)	1.8 (1.2–2.7)	2.1 (1.2–3.6)
Empowerment					
	Yes	128 (71.1)	143 (79.4)	1	
	No	52 (28.9)	37 (20.6)	1.6 (1.0–2.6)	

* Presence of missing data. The SPSS default was used to deal with missing data: a case that has a user-missing value for any variable named is omitted from the computation of the analyses.

Regarding the proximal determinants for dental caries, the majority of individuals in both groups did not regularly use dental services (65.4%), had visited the dentist in the previous year (55.9%), preferred salty foods over sweet (82.0%), consumed sugary foods up to four times a day (93.1%), used dental floss (67.8%) and used toothpaste (97.8%) (Table 2).

**Table 2:** Proximal factors associated with high dental caries in adults, Brazil, 2010.

Proximal factors		Cases (DMFT ≥ 14) n = 180(%)	Controls (DMFT < 14) n = 180(%)	Unadjusted odds ratio (95% CI)	Adjusted odds ratio (95% CI)
**Use of oral health services**					
Regular use *					
	Yes	75 (41.9)	49 (27.4)	1.9 (1.2–3.0)	1.8 (1.1–3.2)
	No	104 (58.1)	130 (72.6)	1	1
Visit to dentist in previous 12 months*					
	Yes	105 (59.0)	93 (52.8)	1	
	No	73 (41.0)	83 (47.2)	0.8 (0.5–1.2)	
**Behavior**					
Food preference *					
	Salty	129 (80.1)	140 (83.8)	1	
	Sweet	32 (19.9)	27 (16.2)	1.3 (0.7–2.3)	
Daily frequency of sugar intake					
	Up to 4 times	163 (90.6)	172 (95.6)	1	
	More than 4 times	17 (9.4)	8 (32.0)	2.2 (0.9–5.3)	
Daily use of dental floss *					
	Yes	127 (71.8)	115 (63.9)	1	
	No	50 (28.2)	65 (36.1)	0.7 (0.5–1.1)	
Use of toothpaste *					
	Yes	158 (96.3)	157 (99.4)	1	
	No	6 (3.7)	1 (0.6)	6.0 (0.7–50.1)	

* Presence of missing data. The SPSS default was used to deal with missing data: a case that has a user-missing value for any variable named is omitted from the computation of the analyses.

In the bivariate conditional logistic regression, dental caries severity was significantly associated with regular visits to the dentist (*p* = 0.004), the type of dental service (*p* = 0.025), petitioning the authorities for community benefits (*p* = 0.009) and monthly household income (*p* = 0.011) (Table 1, Table 2). The distal factor *environmental risk-basic sanitation* was not analyzed because all of the participants reported living in locations with adequate basic sanitation (trash collection and plumbing connected to the public water supply and sewage system).

In addition to the variables with a p value less than 0.05, problems scheduling an appointment (*p* = 0.139), schooling (*p* = 0.077), willingness to dedicate time to community activities (*p* = 0.143), empowerment (*p* = 0.068), daily frequency of sugar intake (*p* = 0.068) and the use of toothpaste (*p* = 0.100) were also tested in the multivariate analysis. Dental caries severity was associated with regular visits to the dentist, income, type of dental service and petitioning the authorities for community benefits. The individuals who regularly visited the dentist had an 80% greater chance of having high caries severity than the remaining individuals (OR: 1.8; 95% CI: 1.1 to 3.2). Those individuals who preferentially used private/supplementary dental services had a 2.3 (95% CI: 1.2 to 4.3) greater chance of having high caries severity than those who used public services more frequently. Individuals with a lower income had a 2.2 (95% CI: 1.3 to 3.9) greater chance of having high caries severity than those with a higher income. Those individuals who reported that the community did not organize in the previous year to petition the authorities for community benefits had a 2.1 (95% CI: 1.2 to 3.6) greater chance of having high caries severity than those who resided in regions in which the authorities were petitioned for community benefits (Table 1, Table 2).

## 4. Discussion

This population-based case-control study in adults identified a higher probability of high dental caries among adults who regularly go to a dentist, use private/supplementary dental services and have low income and low social cohesion. 

The identical variables that were significantly associated with dental caries severity in the bivariate analysis remained significant in the multivariate analysis (regular use of dental services, type of service, income and petitioning the authorities for community benefits). The multivariate analysis and individual matching were important to control for the effect of confounding variables on the DMFT index. While not incorporating any additional variables, this analysis reaffirmed the association of the variables that remained in the final model. 

Both distal (income, petitioning the authorities and type of service) and proximal (regular use of dental services) factors were associated with high dental caries severity (DMFT ≥ 14). Petry *et al.* [16] found a significant association between the regular use of dental services and worse caries status. The regular use of dental services has been demonstrated to be an important factor in a lesser degree of caries severity because this variable is related to social and behavioral factors and is a recognized determinant in the differences regarding caries severity between populations [2,17]. However, in this study, the possibility of reverse causality cannot be eliminated because the greater caries burden most likely led the individuals to seek dental services more frequently. The individuals may have preferred private/supplementary services. The emphasis regarding the care of oral health problems remains centered on restorative treatment, leading to an increase in the number of teeth having undergone some type of clinical intervention [18]. The individuals who visited the dentist more may have received more restorative treatments for factors related to the diagnostic criteria that were used to determine dental caries [16]. It should be stressed that the execution of restorative treatment is an isolated action that does not consider preventive actions aimed at oral conditions [19].

In this study, individuals with a lower income had a greater probability of having high dental caries severity. This finding is in agreement with previous studies that reported an association between poorer oral health status and lower income [5,20,21]. In a previous study [22], the authors concluded that individuals who were unable to afford dental services had a 2.5-fold greater chance of developing new caries than those who were able to afford dental care. This association with dental caries is not limited to differences in household income because income inequity among countries is also associated with this outcome [23].

The probability of having high dental caries severity was greater among the participants who lived in communities that had not organized to petition the authorities for benefits in the previous 12 months. This finding may be explained by the theory that greater social capital aggregates value to the health of individuals, which is reflected in lesser dental caries severity. Social capital is defined as the characteristics of the organization of a society, such as interpersonal trust, norms of reciprocity and support networks. These characteristics capacitate the members of a social group regarding more effective collective actions that are designed to achieve common goals [24]. The lesser caries burden among the participants who lived in communities that petitioned the authorities for benefits underscores the importance of social cohesion in the local formulation of public health policies. The social control proposed by the Brazilian public healthcare system ensures the participation of individuals in decision-making forums [25], which is directly related to the accumulation of social capital by Brazilian society [26]. Community participation provides greater influence in the definition of health priorities. The finding in this study regarding the lesser severity of dental caries (control group) among adults who petitioned the authorities for community benefits underscores the need to consider the effects of social context on health outcome when conducting scientific studies [4,18,24,27].

This study has limitations that should be considered. The study was limited to a segment of the population and territory surrounding the city of Belo Horizonte, and its results are not representative of Brazil. However, measures, such as the random selection of sampling units and the matching of the case and control groups for age and gender, were taken to ensure that the sample was more representative of the region studied. The only variable with a non-response rate higher than 20% was the question concerning an individual's problem scheduling an appointment. Although the DMFT is the most often used index worldwide, it is not sensitive to the effects of social issues on the oral health of a population [28]. A new index, denominated the International Caries Assessment and Detection System, is more specific in the evaluation of the stages of dental caries (white spots through to cavities) [29], but it is not the current WHO model. In this study, the DMFT index was chosen based on its practicality for in-home surveys. There is the possibility of underestimating the number of teeth with carious lesions because the WHO criterion for the diagnosis of dental caries does not involve the use of x-rays, hindering the detection of hidden and interproximal caries. We used the total DMFT based on the WHO evaluation of the severity of dental caries [3]. Because the DMFT index is largely affected by treatment history, older age could be a confounding factor in the greater severity of dental caries. Matching by gender was important because in-home studies tend to have greater participation by women, who are more likely to be found in the residence. The sex difference in oral health has been documented over time and across cultures. The oral health of women declines more rapidly than that of men [30]. The total DMFT index score in adults reflects the long-term effects of prevention and treatment policies. It is necessary to evaluate the determinants of each component of the DMFT in other studies. Because of the case-control design, the associations between greater dental caries severity and the final model variables cannot be interpreted in terms of cause and effect.

Despite these limitations, this study is important because it is the first case-control study to assess the severity of dental caries in adults in the investigated region. After the findings of the cross-sectional study [6], the associations identified in this case-control study could be useful in the development of prospective cohort studies. It is important to emphasize that the case-control methodology provides better information about determinants, comparing to cross-sectional approach [1]. Besides, the number of independent variables was larger than in the previous study [6]. The results of this study underscore the importance of considering the distal and proximal factors in the evaluation of the severity of dental caries because variables from both of these categories remained associated with the outcome after controlling for the confounding factors. The proximal factors of health behavior considered to be protective, such as proper oral hygiene and dietary habits, were not associated with lesser dental caries severity. Socio-cultural determinants, income and social support networks may explain the inequalities in the rates of dental caries severity among adults. The findings demonstrate that greater dental caries severity persists among individuals with a lower household income and among groups with lesser social cohesion because the social context aspects had a statistically significant effect on the severity of dental caries. Thus, greater or lesser social cohesion in a community can play an important role in the differences in dental caries severity, emphasizing the need for intersectoral interventions with a public health perspective. Future prospective cohort studies on dental caries among adults with similar social conditions could use the variables identified in this case-control study as exposures.

## 5. Conclusions

The results of the study underscore the importance of considering distal and proximal factors in the assessment of the severity of dental caries. A greater degree of caries severity persists among low-income families and among groups with a low degree of social cohesion.
